# Analysis of Psychosocial Adjustment in the Family During Confinement: Problems and Habits of Children and Youth and Parental Stress and Resilience

**DOI:** 10.3389/fpsyg.2021.647645

**Published:** 2021-07-15

**Authors:** Magdalena P. Andrés-Romero, Juan M. Flujas-Contreras, Mercedes Fernández-Torres, Inmaculada Gómez-Becerra, Pilar Sánchez-López

**Affiliations:** ^1^Department of Psychology, University of Almería, Almería, Spain; ^2^Health Research Center (CEINSA/UAL), University of Almería, Almería, Spain

**Keywords:** adolescence, COVID-19, children, habits, parents, parental stress, parental resilience, lockdown

## Abstract

The COVID-19 health crisis has led to a dramatic change in dynamics and habits of families, which may be a factor involved in the development and maintenance of problems and difficulties in children. The present study is a cross-sectional study that aims to describe and analyze the relationship between the difficulties in psychological adjustment and the change of habits of the infant-juvenile population as perceived by their parents and their stress and resilience during the total confinement of the first wave of the COVID-19 pandemic in Spain, as well as analyzing the course of the changes and the relationships between weeks 3 to 6, that is, the score of different participants in each week of the confinement. The sample is comprised of 883 parents of children and adolescents between 3 and 18 years of age. Children’s psychological adjustment, children’s habits, parental stress, and parental resilience were assessed by parents. The results show that parents perceive a change in the habits and psychological difficulties in their children. At the same time, our results describe parents with a high level of stress and resilience, with differences depending on the children’s ages. The time of confinement accentuates the perception of parents about the psychological difficulties of their children and parental stress, as well as a decrease in resilience. These difficulties are reduced when the parent has resilience competencies. These results show that the resilience of parents mediate the relationship between parental stress and psychological problems of their children. These results shows that COVID-19 lockdown had a considerable effect on families, both on children and parents. Some practical implications based on results are provided.

## Introduction

In order to contain and mitigate COVID-19, countries have adopted different health, social and education measures (Imai et al., 2020). Among others, home confinement and school closures have affected 86% of the children around the world, propitiating changes in general social functioning and increased inequality (The Lancet Child Adolescent Health, 2020). The need to maintain epidemiological control of the pandemic has entailed a drastic change in the lives of citizens in all the areas of life, from work to home, altering daily practices and family habits. However, what from the strictly healthcare viewpoint seems an ineludible demand, can have implications for children which cannot be ignored. It is important to maintain certain habits, especially in childhood and adolescence, because of the cognitive repercussions (Maureira Cid and Flores Ferro, 2017; Pisch et al., 2019), on acquisition of behavior patterns and social knowledge, children’s health and social and economic benefits to their development and that of future generations (World Health Organization [WHO], 2020). It is easily surmised that such government measures have affected the routines of both adults (Liu N. et al., 2020; Sun et al., 2020) and children (Berasategui Sancho et al., 2020; Orgilés et al., 2020), although the significance of the change in routines and the psychological impact that this could have on parents and children is hard to define. As already demonstrated, it is influenced by its duration, and other factors, such as fear, inadequate information or modification in the daily lives of children (Brooks et al., 2020; Gómez-Becerra et al., 2020; Sandín et al., 2020). The significance of changes in habits of small children after traumatic events was described by Echeburúa et al. (2004) as the more problematic expression of sorrow, exteriorized by a loss of learning and acquired habits, withdrawal, fear, unexpected reactions, accentuation of preexisting traits (nervousness or sadness) as well as separation anxiety. Thus, sleep problems have been shown to be predictors of separation anxiety (Orgilés et al., 2016), which is especially important considering the vulnerability of children who have been separated from their caregivers during the pandemic (Liu J. J. et al., 2020). Studies focused on the effects of continual exposure to violence or terrorism, have observed that short-term changes produce long-term collective habituation in children and adults, as well as development of strengths (hope, optimism, gratitude, spirituality or altruism, curiosity, among others) in both individuals and groups (Vázquez et al., 2008).

According to the classic definition of stress (Folkman and Lazarus, 1985; Lazarus and Folkman, 1986) as a phenomenon that appears when a situation overwhelms personal resources and coping strategies are insufficient or inadequate, the current situation seems to meet all the requirements for the appearance of such a response in parents. School closures have been an added burden for parents who must take on the school routines of their children along with other demands and concerns (emotional, hygiene, work, economic, etc.) the pandemic has brought on. This work overload of the main caregiver has been related to parental stress Vela Llauradó and Suárez Riveiro, 2020), and in turn, with competencies and attitudes in the role of parent (Pérez Padilla et al., 2010).

In the family context, birth of children and bringing them up, which leads to personal restructuring, assuming responsibilities or discrepancy between expectations for parenting and its real demands, have been considered potentially stressful events, which has led to giving parental stress a definition with its own entity (Cameron et al., 1991; Berry and Jones, 1995). Although it has conceptual differences from other forms of stress, it shows close association with them (Holly et al., 2019). Deater-Deckard (2004) defined parental stress as “a set of processes that lead to adverse psychological and physiological reactions derived from the attempts of adapting to the demands of parenting. This is often experienced as negative feelings or beliefs about oneself and the child” (p.6), to which environmental and social circumstances should be added (Cronin and Becher, 2015).

However, not all families experience the same amount of stress, as their approach to parenting may differ, and personal characteristics of both adults and children, family climate and socioeconomic setting of the family nucleus (see Hayes and Watson, 2013 for a review) all strongly influence the demands on parents (Oronoz et al., 2007), their perception of their competence and self-efficacy as parents (Raikes and Thompson, 2005; Díaz-Herrero et al., 2010), as well as paternal, family (Ispa et al., 2004; Putnick et al., 2008; Estes et al., 2009; Farmer and Lee, 2011) and children’s characteristics (Byrne and Cunningham, 1985; Anastopoulos et al., 1992; Crnic and Low, 2002; Gupta, 2007; Tripp et al., 2007; Wang et al., 2011; Mount and Dillon, 2014; Kim, 2017; Rajan and John, 2017).

Studies analyzing social factors that could be determinant in parenting, have found that parental stress is more prevalent in families in a vulnerable situation (Ceballo and McLoyd, 2002; Chaudry and Wimer, 2016). Factors commonly associated with this vulnerability are low parent education (Raikes and Thompson, 2005), single parenting (Olhaberry and Farkas, 2012), economic problems, unemployment and low-qualification jobs (Duncan and Brooks-Gunn, 2000; Ayala-Nunes et al., 2014). These also seem to be facilitators not only of parental stress, but of low self-perception of competence as parents (Raikes and Thompson, 2005; Morrison et al., 2014).

One psychological phenomenon frequently studied with regard to stress is resilience, whether understood as an individual trait or personality attribute, as a result of the person’s behavior, or as a dynamic process (García del Castillo et al., 2016), enabling a healthy response to adversity or possible stressors (Ortega, 2014). Theoretical-practical interest in this construct has not stopped growing since the first studies in the last decades of the 20th century (Villalba Quesada, 2003; Becoña, 2006; Wagnild, 2009; Pinto-Cortez, 2014; Fernandes de Araújo et al., 2015; García del Castillo et al., 2016; Ramón Fernández et al., 2019).

Studies defining the relationship between resilience and other variables have demonstrated its buffering effect in people exposed to adverse situations, such as adults exposed to armed conflict in Colombia (Hewitt Ramírez et al., 2016), who have to struggle with stress, anxiety and/or depression (Bitsika et al., 2013). In fact, the positive correlation between resilience and psychological wellbeing, activities promoting health, sense of life, coherence, morality and age has been underlined. In comparison, there is a negative correlation between resilience and stress, anxiety and/or depression (Wagnild, 2009; Sánchez-Teruel and Robles-Bello, 2015). Along this line, Fernandes de Araújo et al. (2015) emphasized the role of resilience in the family as a protective factor and mediator in coping with negative circumstances, stressing the association between high levels of resilience and little presence or manifestation of psychological disorders.

In our review of the literature, we already found studies evaluating resilience in coping with the COVID-19 crisis (Giallonardo et al., 2020; Lozano-Díaz et al., 2020; among others*)*. However, it has become necessary to go more deeply into resilience as it relates to stress, and specifically, parental stress, in view of the exceptional circumstances experienced by families as a consequence of the lockdown and social distancing imposed to contain the disease.

The relationship between parental stress and resilience has been widely documented in families with children who have neurodevelopmental disorders, and in such families, parents usually have more stress and less resilience than those with children with no disorder (Cara García, 2019). In that line, it has been observed that the parents of children with Autistic Spectrum Disorder (ASD) have scored significantly lower in resilience than parents of children with normal/typical development (Tijeras Iborra, 2017). In a study with a sample of 299 families with children with and without disability, Vela Llauradó and Suárez Riveiro (2020) defined the relationship between parental stress and resilience as significant and negative, and considered the latter as a protective factor against stress. Supporting this relationship, Suzuki et al. (2018) stated that family resilience reduces maternal stress, in as much as the capacity for resilience in the family influences individual resilience.

The present cross-sectional study described and analyzed the relationship between the difficulties in psychological adjustment and changes in habits of the infant-juvenile population as perceived by their parents, and parental stress and resilience during total confinement due to the first wave of the COVID-19 pandemic in Spain. It also analyzed family response by the week of confinement when they filled in the questionnaire, from weeks three to six. The following specific objectives were pursued:

(1) Describe the variables studied (children’s problems and changes in habits, as well as parental stress and resilience) both in the total sample and by children’s age/grade level and by week of confinement.
(2) Find out whether there were significant differences in the parents’ perception of their children’s psychological problems and changes in their habits, as well as self-perceived stress and resilience, by children’s ages and/or week of confinement.
(3) Explore the relationship between perceived children’s psychological problems and changes in habits and parental factors (stress and resilience).
(4) Assess the influence of parents’ perceived changes in their children’s habits on both perceived children’s psychological problems and parental factors (stress and resilience).
(5) Examine the role of resilience between parents’ self-perceived stress and their perception of their children’s psychological problems.

In light of the information reviewed, the following hypotheses were proposed:

Hypothesis 1: The psychological adjustment of parents, children and adolescents will be low, that is, problems will be perceived in children and parental stress will be above the mean.

Hypothesis 2: Children’s psychological adjustment problems will increase with time of confinement. Families that have been confined fewer weeks will perceive fewer children’s psychological problems than families that have been confined longer.

Hypothesis 3: There will be significant differences in children’s adjustment and habits, and parental stress and resilience by week of confinement and age group.

Hypothesis 4: Parents with higher stress will perceive more psychological difficulties in their children.

Hypothesis 5: Parents who perceive themselves to be more resilient will be less stressed.

Hypothesis 6: Parents who perceive changes in their children’s habits will report that their children have more psychological problems and will themselves have higher levels of stress.

Hypothesis 7: Resilience will mediate between parental stress and children’s perception of psychological difficulties.

## Materials and Methods

### Participants

The reference population for this study was made up of parents with children aged 3 to 18. Exclusion criteria were a child’s clinical diagnosis of disability, psychological disorder or severe illness. With this target population, the original sample surveyed was 1078 families. When the incomplete or invalid surveys had been eliminated, the final sample was 883 parents aged 25 to 69 (*M* = 40.46; *SD* = 6.16). Mostly mothers answered (93.9%), almost all were Spanish (95.6%), and most had a high school or university education (74.4%). The sample was mostly employed in services (45.2%) or in education, culture and health (37.7%). These data distributed by age of their children is shown in Table 1. 81.8% of the participants said there were two parents in the family. The most frequent employment situations of the sample during lockdown were working online due to COVID-19 (32%) and dismissal due to the crisis generated by the pandemic (20.2%) and two conditions that did not undergo change as a consequence of the crisis, unemployed before and now (13.8%) and going to work before and now (13.8%).

**TABLE 1:** Sociodemographic data of sample (*N* = 883).

**Children’s Age range/**	**% of**	**Age of parents.**	**% of**	**% of Biparental**	**% of secondary studies**
**Edutation levels**	**mothers**	**M(SD)**	**Spanish**	**families structure**	**or more (parents)**
Toddlers - less than 3 years (*n* = 65)	98.5	35.74 (4.11)	100	90.8	78.5
Toddlers - 3 to 5 years (*n* = 283)	94.7	36.8 (4.51)	94	89	76.3
Basic education (6 to 11 years) (*n* = 353)	95.8	40.84 (4.95)	95.4	78.9	72.8
High school (12 to16 years) (*n* = 122)	90.2	45.68 (4.8)	96.7	73	68
High school (more than 16 years) (*n* = 60)	81.7	50.43 (3.86)	96.7	73.3	83.3

Most of the families (53.1%) had two children, 36.1% had one child, and 9.7% had three children, and only 1% had four or more children. Considering the total number of children in the sample (*n* = 1555), the majority of children are female (53.18%; *n* = 827) compared to 46.81% (*n* = 728) who are male. The ages of children were distributed as illustrated in Table 1.

### Study Type and Design

The study was a cross-sectional description of populations, conducted by means of a survey with probabilistic samples to acquire empirical evidence using a quantitative methodology (Montero and León, 2007).

The study started in the third week of confinement until the sixth week, when the children were allowed to leave home again; therefore, data collection lasted 4 weeks. The study, even with a cross-sectional methodology, allowed us to evaluate the weekly changes throughout the confinement by making comparisons between the questionnaires collected over the weeks. At the same time, in all cases the context of data collection was online with a non-probabilistic ”snowball” sampling strategy.

### Instruments and Variables

The variables selected for this study analyzed in families through a battery of online questionnaires, were the following:

Sociodemographic variables of the parents, measured using questions prepared for the purpose at the start of the survey.

General psychological state of children and adolescents with the Spanish version of the *Strengths and Difficulties Questionnaire* (SDQ; Goodman, 1997). This instrument is a dimensional measure of mental health and screening for emotional and behavior problems. The questionnaire is applied to parents, teachers and/or adolescents. It is comprised of 25 items rated on a three-point Likert-type scale and distributed in five subscales: emotional symptoms, behavior problems, hyperactivity, peer relations problems and prosocial behavior. Its psychometric properties are internal consistency of α = 0.79. This study used the reported version for parents, which has an internal consistency of.58 to.80 (Español-Martín et al., 2020). The scales established were followed with cutoff points at the 80th percentile for subclinical and 90th for clinical ranges, except for the prosocial behavior subscale, which is at the 10th and 20th percentile, respectively (Español-Martín et al., 2020). Internal consistency in this study was a Cronbach’s alpha of.74 for the total score. For the subscales, the Cronbach’s alpha was.54 for the emotional symptoms scale, 0.59 for behavior problems, 0.76 for the hyperactivity scale, 0.57 for problems with peers and.38 for prosocial behavior.

A self-report designed for the purpose on *habits of everyday living* and possible changes that may have occurred in them during lockdown, comprised of seven items with a five-point Likert scale (1 = not at all to 5 = very much), which evaluate sleep routines, eating patterns, hygiene habits, interpersonal relations with friends and family, emotional state, and fear, with an internal consistency of α = 0.67 (Appendix 1 shows the specific items of the self-report).

The level of parental stress was evaluated using the Spanish version (Oronoz et al., 2007) of the *Parental Stress Scale* (PSS) by Berry and Jones (1995) is composed of 12 items on a 5-point Likert scale and assesses stress in two sub-scales: (1) child rewards, which refers to satisfaction as a parent in your parental role; and (2) stressors, which refers to the level of stress related to parenting. A higher score means a higher level of stress, the mean score in the total test is for women 22.3 and for men 20, in direct scores; and for the sub-scales the means are: in reinforcers for women 6.9 and for men 7.1; while for stressors the mean for women is 15 and for men 13. The internal consistency of the scale is a Cronbach’s alpha of.77 for the rewards subscale and.76 for the stressors subscale.

Parental capacity for resilience was measured with the Spanish version of the 14-item *Resilience Scale (ER-14)* by Wagnild (2009). It considers resilience a positive personality characteristic which enables individual adaptation to adverse situations. The RS-14 measures two factors: (1) Personal competence which is composed of 11 items assessing self-confidence, independence, decisiveness, resourcefulness, and perseverance, and (2) the self-acceptance scale, which is composed of three items assessing adaptability, balance, flexibility, and a stable life perspective. The responses are scored on a Likert scale from 1 to 7. The mean scale score is 71 (SD = 32.81). For the subscales it is 56 (SD = 19.29) and 15.08 (SD = 7.84), respectively. The version of the scale by Sánchez-Teruel and Robles-Bello (2015) shows adequate internal consistency (α = 0.79) and validity of criteria calculated with other measures of general resilience (CD-RISC) (*r* = 0.87; *p* < 0.01).

### Procedure

First, the survey or battery was designed. Tests for this had previously been selected according to their contents on the variables of interest and psychometric properties. These were transferred to an online evaluation protocol using *LimeSurvey*^®^.

After requesting the authors’ university bioethics committee for the pertinent authorizations, the test was published in three ways: (1) An incidental sample through social networks. (2) A convenience sample acquired with distribution focused on various professions that work with families. (3) Interprofessional relationships in the sectors mentioned in the second group above, who were contacted by email.

Furthermore, it should be noted that the socio-educational context at the time of the study in Spain was characterized by total confinement, in which children and adolescents could not attend school, go out to the parks, go to the streets, or participate in their usual leisure activities. They could not visit other family members or friends. Their parents, in turn, could be teleworking or unemployed. In many families there could be sick, isolated or even hospitalized family members. All of these restrictions were drastic, and were not clearly established from the outset, but were updated weekly, so there was a degree of uncertainty regarding when the restrictions would end.

Finally, the participants answered after reading all ethical aspects and guarantees and giving their informed consent.

### Data Analysis

First, a descriptive analysis of the variables (mean and standard deviation) was done for the total sample and by age and grade level (under 3 years (1st cycle toddlers), from 3 to 5 (2nd cycle toddlers), from 6 to 11 (basic education), from 12 to 15 (high school) and 16 to 18 (high school). Differences between age groups were evaluated by one-factor ANOVA. Differences between participants who completed the assessment from the third to sixth week were studied, with a one-factor ANOVA in which the week of response was the comparison factor between variables analyzed. Furthermore, to examine the effects of time of confinement based on age range, a MANOVA was done in which the time^∗^age interaction was considered. The effect size for the analysis of variance was performed using etha-square. *Post hoc* comparisons were performed, and the effect size was estimated using Cohens’ d. Relationships between variables analyzed were explored with a Pearson correlation analysis.

The differences in parenting variables (stress and resilience) and children’s problems were evaluated by perception of change in habits during lockdown. The Student’s *t* for independent samples was used to compare the parents’ mean scores. A score below 3 indicated little change in habits, while a score over 3 showed sizeable or significant change. The effect size for was determined by using the Cohens’ d.

A multiple regression analysis was done, in which the total difficulties score (SDQ) as the dependent variable and changes in children’s habits as the independent variable, to find out which variables explained the variance in change in children’s habits. Finally, a mediation analysis was done of parental stress and the variables of difficulties evaluated with the SDQ, in which own personal resilience and acceptance of oneself were considered mediators. The analyses were done using SPSS 21. The mediation analysis was done with the R program.

## Results

### Descriptive Analysis and Role of Age/Educational Level and/or Week of Confinement

Table 2 shows the mean scores and standard deviations of the variables. The mean scores on the SDQ were within the limits for emotional symptoms, behavior problems, hyperactivity/inattention, prosocial behavior and total SDQ difficulties score. The average parental stress scores were above the normative mean of the evaluation instrument. The resilience scores were above the mean on the personal competence scales and the total for the instrument, although the mean score on acceptance of oneself on resilience was below the mean. The mean score in changes in habits was 17.8 (*SD* = 4.53) which is 6.11 points above the mean between the instrument maximum and minimum.

**TABLE 2:** Mean scores and standard deviation of variables for the total sample (*N* = 883), by school levels and differences in means by age ranges.

	**Total simple (*n* = 883)**		**Years (scholar level)**										**ANOVA**		
			**Toddlers - less than 3 years (*n* = 65)**		**Toddlers - 3 to 5 years (*n* = 283)**		**Basic education (6 to 11 years) (*n* = 353)**		**High school (12 to16 years)**		**High school (more than 16 years)**				
	***M***	***DT***	***M***	***DT***	***M***	***DT***	***M***	***DT***	***M***	***DT***	***M***	***DT***	***F***	***p***	***n*^2^**
SDQ	13.00	5.47	14.92	4.68	13.65	5.14	13.26	5.59	11.12	5.71	10.18	4.81	11.25	0.000	0.049
Emotional Sx.	3.69	1.81	3.82	2.04	3.56	1.70	3.91	1.83	3.39	1.88	3.50	1.74	2.69	0.03	0.012
Behavioral Px.	2.40	1.66	2.88	1.73	2.61	1.63	2.42	1.62	1.97	1.72	1.55	1.38	8.796	0.000	0.039
Hyperactivity	5.21	2.53	6.45	2.33	5.85	2.35	5.19	2.53	3.97	2.23	3.43	2.28	25.72	0.000	0.105
Peer Px.	1.71	1.65	1.78	1.47	1.62	1.52	1.74	1.78	1.80	1.74	1.70	1.49	0.343	0.849	0.002
Pro social	6.15	1.67	5.74	1.75	6.05	1.64	6.40	1.69	5.98	1.61	5.88	1.54	4.016	0.003	0.018
Routines	17.78	4.53	19.20	4.67	18.08	4.41	17.72	4.51	17.32	4.54	16.17	4.57	4.206	0.002	0.019
PSS	25.96	7.91	28.89	6.56	26.20	7.67	26.31	7.66	24.57	8.95	22.40	8.20	6.598	0.000	0.029
Stressors	18.83	6.57	21.72	5.48	19.16	6.24	19.21	6.48	17.21	7.08	15.25	6.70	10.33	0.000	0.045
Rewards	7.12	2.75	7.17	2.34	7.04	2.59	7.10	2.77	7.36	3.32	7.15	2.52	0.306	0.874	0.001
R-14	75.11	11.73	70.28	12.68	74.39	11.91	75.36	11.51	77.44	11.38	77.50	10.19	4.983	0.001	0.022
Competence	60.62	9.20	57.28	9.48	60.08	9.40	60.87	9.12	62.14	8.79	62.25	8.27	3.801	0.005	0.017
Acceptance	14.48	3.20	13.00	3.72	14.30	3.21	14.49	3.06	15.30	3.14	15.25	2.85	6.766	0.000	0.03

*Sx.: symptoms; Px.: problems.*

**FIGURE 1:**
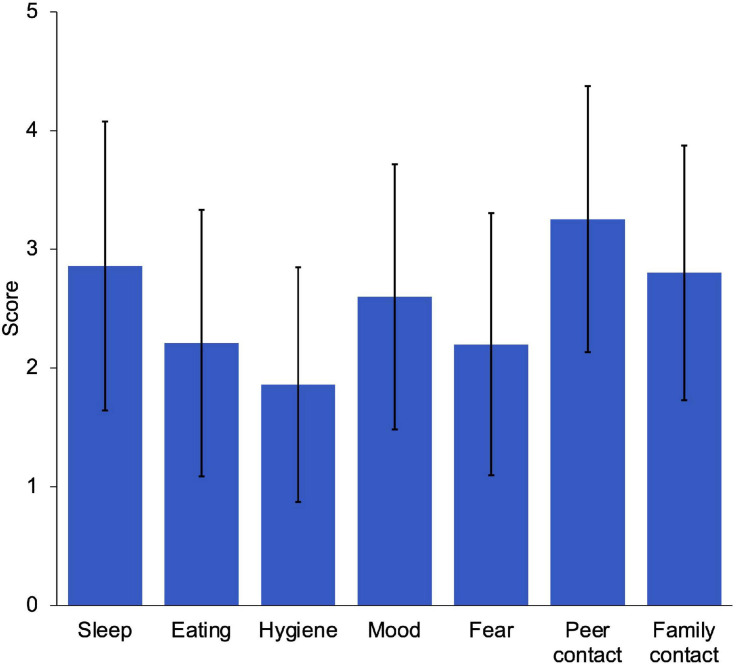
Mean scores and standard deviation in change of children’s habits.

Statistically significant scores were found by age (divided into grade levels) in total SDQ difficulties, emotional symptoms, behavior problems, hyperactivity symptoms and prosocial behavior. Statistically significant differences were found for parents in stress, stressors, resilience, personal competence and acceptance of oneself. Statistically significant differences were found in habits between age ranges (Table 2). In addition, it may be observed how family members reported the difficulty in contact with peers (*M* = 3.25; *SD* = 1.12), sleep problems (*M* = 2.86; *SD* = 1.21) and contact with family members (*M* = 2.8; *SD* = 1.07) were the most frequent changes in their children’s habits (Figure 1).

In relation to the differences between age groups, in the *post hoc* analysis we found statistically significant differences in the total score of difficulties (SDQ) between children under 3 years of age with children between 12 and 16 (*t* = 4.465; *p* < 0.001; *d* = 0.7) and with children older than 16 years (*t* = 4.948; *p* < 0.001; *d* = 1). On the other hand, we found these differences between the scores of children between 3 and 6 years old with children between 12 and 16 (*t* = 4.355; *p* < 0.001; *d* = 0.45) and with children older than 16 (*t* = 4.554; *p* < 0.001; *d* = 0.68). As well as, statistically significant differences were found among children between 6 and 11 years of age with children between 12 and 16 (*t* = 3.799; *p* < 0.005; *d* = 0.38) and with children older than 16 (*t* = 4-115; *p* < 0.001; *d* = 0.56). Significant differences were found in the score of the behavioral problems scale between children aged older than 16 years compared to children under 3 years (*t* = 4.539; *p* < 0.001; *d* = 0.84), children between 3 and 6 years (*t* = 4.673; *p* < 0.001; *d* = 0.66) and children between 7 and 11 years (*t* = 3.837; *p* < 0.001; *d* = 0.55). In the hyperactivity problems scale, differences were found between children under three years of age compared to the ranges of 6 to 11 years (*t* = 3.883; *p* < 0.001; *d* = 0.5), 12 to 16 years (*t* = 6.719; *p* < 0.001; *d* = 1.09) and older than 16 years (*t* = 7.004; *p* < 0.001; *d* = 1.308). There were differences between children between 3 and 6 years of age with 6 to 11 years (*t* = 3.467; *p* < 0.01; *d* = 0.27), 12 to 16 years (*t* = 7.242; *p* < 0.001; *d* = 0.81) and older than 16 years (*t* = 7.082; *p* < 0.001; *d* = 1.034). In this scale, differences are found between children between 6 and 11 years old with children from 12 to 16 years old (*t* = 4.834; *p* < 0.001; *d* = 0.46) and older than 16 years old (*t* = 5.227; *p* < 0.001; *d* = 7). No significant differences were found in the rest of the *post hoc* comparisons of the SDQ scales.

Regarding parental stress, differences were found between parents of children under 3 years of age compared to parents of children over 16 years of age (*t* = 4.64; *p* < 0.001; *d* = 0.87). Likewise, differences were found between parents of children between 3 and 6 years of age compared to parents of children over 16 years of age (*t* = 3.419; *p* < 0.001; *d* = 0.48). Differences are found between parents of children between 7 and 11 years old compared to parents of children older than 16 years old (*t* = 3.579; *p* < 0.001; *d* = 0.5).

In relation to parental resilience, differences were found between parents of children under 3 years old compared to parents of children between 12 and 16 years old (*t* = 4.013; *p* < 0.001; *d* = 62) and parents of children over 16 years old (*t* = 3.469; *p* < 0.001; *d* = 0.62).

In order to check for changes in parents’ perceptions between weeks of confinement an ANOVA analysis is performed. Table 3 shows that there were statistically significant differences in the scores reported by the parents in the different weeks during lockdown in all the variables except for prosocial behavior and rewards of parenting. As the weeks of lockdown went on, higher scores were observed on the SDQ scales, that is, more perception of children’s problems and difficulties. In problems with peers, variability was observed between weeks and prosocial behavior remained stable. For the hyperactivity or inattention score and SDQ overall score the effect size is moderate (see Table 3 for effect sizes). Changes in habits increased as the weeks of lockdown went on, with a moderate effect size. Regarding, the instruments referring to the parents showed a certain homogeneity in the rewards of parenting scores over the weeks and instability in stress, although the scores in the last week were higher than in the first. Resilience was observed to decrease over the weeks, dropping more noticeably in acceptance of oneself. For these variables a small effect size is observed.

**TABLE 3:** Mean scores, standard deviation and differences in points of assessment.

	**3rd week**		**4th week**		**5th week**		**6th week**		**ANOVA**		
	***M***	***SD***	***M***	***SD***	***M***	***SD***	***M***	***SD***	***F***	***p***	**η*^2^***
SDQ	11.14	4.90	13.53	5.27	12.42	5.31	15.08	5.59	25.92	0.00	0.081
Emotional Sx.	3.25	1.61	3.81	1.76	3.59	1.83	4.17	1.93	12.00	0.00	0.039
Behavioral Px.	1.98	1.51	2.53	1.63	2.27	1.68	2.84	1.72	12.90	0.00	0.042
Hyperactivity	4.44	2.51	5.32	2.30	5.02	2.43	6.11	2.51	20.67	0.00	0.066
Peer Px.	1.47	1.47	1.88	1.85	1.55	1.54	1.97	1.72	5.29	0.00	0.018
Pro social	6.00	1.73	6.11	1.63	6.26	1.57	6.26	1.69	1.35	0.26	0.005
Change habits	16.37	4.24	17.73	3.93	17.59	4.40	19.55	4.80	23.39	0.00	0.074
PSS	25.18	8.23	27.09	7.48	25.28	7.41	26.40	8.11	2.91	0.03	0.011
Stressors	18.15	6.78	19.94	6.38	18.35	6.27	19.07	6.59	3.24	0.02	0.011
Rewards	7.03	2.73	7.16	2.28	6.94	2.63	7.34	3.15	0.87	0.46	0.003
R-14	76.72	10.73	74.37	11.80	75.69	11.37	73.47	12.76	3.79	0.01	0.013
Competence	61.66	8.54	59.89	9.23	60.92	8.70	59.83	10.09	2.27	0.08	0.008
Acceptance	15.06	2.83	14.48	3.20	14.77	3.16	13.64	3.45	9.51	0.00	0.031

*Sx: symptoms; Px.: problems.*

*Post hoc* analyses showed significant differences in emotional symptoms between the third week of confinement and the fourth (*t* = −3.333; *p* < 0.05; *d* = 0.332) and sixth week (*t* = −5.883; *p* < 0.001; *d* = 0.517), as well as between the fifth and sixth week (*t* = 3.229; *p* < 0.05; *d* = 0.305). In the behavioral problems scale, significant differences were found between the third week of confinement and the fourth (*t* = −3.561; *p* < 0.005; *d* = 0.348) and sixth week (*t* = −6-043; *p* < 0.001; *d* = 0.553), as well as between the fifth and sixth week (*t* = −3.476; *p* < 0.005; *d* = 0.335). Hyperactivity symptoms show statistically significant differences between the third week with the fourth (*t* = −3.850; *p* < 0.001; *d* = 0.365) and sixth (*t* = −7.783; *p* < 0.001; *d* = 0.664). Differences were also found between the fourth (*t* = −3.315; *p* < 0.05; *d* = 0.323) and fifth (*t* = −4.408; *p* < 0.001; *d* = 0.438) weeks with the sixth week of confinement. For the peer problems scale, significant differences were found between the third week compared to the fourth (*t* = −2.659; *p* < 0.05; *d* = 25) and sixth week (*t* = −3.501; *p* < 0.005; *d* = 0.315). Regarding the overall SDQ score, differences are observed between the third week with the fourth (*t* = −4.86; *p* < 0.001; *d* = 0.474) and sixth (*t* = −8.591; *p* < 0.001; *d* = 0.754). Differences are also found between the fourth (*t* = −3.065; *p* < 0.05; *d* = 0.284) and fifth (*t* = −5.028; *p* < 0.001; *d* = 0.485) week with the sixth week of confinement. For all these variables the effect size is greater for the differences between the third and sixth week, although with a moderate effect size.

Regarding changes in habits, statistically significant differences were observed between the sixth week of confinement with the third (*t* = −8.345; *p* < 0.001; *d* = 0.707), fourth (*t* = −4.33; *p* < 0.001; *d* = 0.410) and fifth (*t* = −4.447; *p* < 0.001; *d* = 0.424) week.

Regarding parental stress scores, statistically significant differences were found between the third and sixth week (*t* = −2.593; *p* < 0.05; *d* = 0.242). As for resilience scores, differences were observed in the self-acceptance scale between the third and sixth week (*t* = −5.172; *p* < 0.001; *d* = 0.454). Similarly, the overall resilience score showed significant differences between the third and sixth weeks (*t* = 3.19; *p* < 0.005; *d* = 0.278).

In order to determine the differences according to age and weeks of confinement a MANOVA was done using points of assessment (scores of different participants in 3rd, 4th, 5th, and 6th week of lockdown) and age by grade level as factors to evaluate the effects of lockdown on the time^∗^children’s age relationship in all the assessed variables. A significant effect in this interaction was observed in hyperactivity symptoms evaluated with the SDQ (*F* (12) = 1.807; *p* < 0.05; η*^2^* = 0.25), with a small effect size. In particular, *post hoc* analysis show that this interaction was significant in children between three-to-six years between the fifth and sixth week (*t* = 1.722; *p* < 0.001). In children between 7-to-11 years, significant differences were found between the third week and fourth (*t* = 1.089; *p* < 0.005), fifth (*t* = 1.327; *p* < 0.005) and sixth weeks (*t* = 1.516; *p* < 0.001). No statistically significant differences were found in the rest of the variables.

### Correlational Analysis

Table 4 shows statistically significant bivariate correlations between most of the variables. There was no statistically significant correlation between the total change in habits and prosocial behavior scores. The relationships between the parents’ personal resilience scores, both total and on subscales, were negative and statistically significant with the rest of variables, except for prosocial behavior, in which the correlation was positive.

**TABLE 4:** Pearson’s bivariate correlations.

	**PSS**	**Stressors**	**Rewards**	**R-14**	**Competence**	**Acceptance**	**Routines**
SDQ	0.477**	0.462**	0.268**	−0.273**	−0.224**	−0.358**	0.520**
Emotional Sx.	0.275**	0.285**	0.112**	−0.139**	−0.099**	−0.225**	0.389**
Behavioral Px.	0.450**	0.421**	0.289**	−0.262**	−0.227**	−0.309**	0.446**
Hyperactivity	0.371**	0.375**	0.171**	−0.208**	−0.163**	−0.293**	0.392**
Peer Px.	0.256**	0.221**	0.211**	−0.170**	−0.155**	−0.178**	0.246**
Pro social	−0.175**	−0.115**	−0.229**	0.204**	0.226**	0.098**	0
PSS		0.945**	0.621**	−0.363**	−0.338**	−0.358**	0.307**
Stressors			0.329**	−0.250**	−0.223**	−0.277**	0.283**
Rewards				−0.446**	−0.441**	−0.370**	0.205**
R-14					0.982**	0.844**	−0.231**
Competence						0.728**	−0.210**
Acceptance							−0.242**

Sx.: symptoms; Px.: problems; **: *p* < 0.001.

### Effects of Changes in Habits on the Variables and Linear Regression Analysis

Table 5 illustrates the differences in mean scores and effect sizes of the variables by perception of change in children’s habits. Thus, statistically significant differences were found for most of the variables depending on which of the habits was evaluated. Those who reported changes in their children’s habits scored higher on difficulties in children’s behavior and parental stress. The mean scores in personal competencies of resilience and acceptance of oneself were lower for those who reported more change in habits. The prosocial behavior variable showed statistically significant differences only with change in fears, contact with peers and contact with family members, in which the mean score was low for children of parents who reported changes in those respects.

**TABLE 5a:** Mean scores, standard deviation and Student t to evaluate the differences in the variables according to the perception of habit change in the children.

		**Sleep habits**				**Eating habits**				**Hygiene habits**				**Change in mood**			
		***M***	***SD***	***t***	***d***	***M***	***SD***	***t***	***d***	***M***	***SD***	***t***	***d***	***M***	***SD***	***t***	***d***
SDQ	*No change*	10.78	4.71	−10.3*	0.712	11.63	5.00	−10.2*	0.712	12.40	5.26	−6.11*	0.488	10.60	4.53	−14.3*	0.966
	*Change*	14.46	5.45			15.31	5.47			15.01	5.69			15.36	5.29		
Emotional Sx.	*No change*	3.17	1.62	−7.11*	0.489	3.32	1.62	−8.14*	0.567	3.55	1.73	−4.14*	0.33	3.11	1.51	−9.96*	0.670
	*Change*	4.03	1.86			4.31	1.95			4.15	1.99			4.26	1.91		
Behavioral Px.	*No change*	1.81	1.41	−8.93*	0.614	2.05	1.51	−8.21*	0.571	2.21	1.57	−6.15*	0.491	1.69	1.34	−13.8*	0.929
	*Change*	2.78	1.70			2.97	1.74			3.01	1.81			3.09	1.66		
Hyperactivity	*No change*	4.35	2.36	−8.46*	0.582	4.73	2.50	−7.40*	0.515	5.03	2.53	−3.85*	0.307	4.30	2.37	−11.2*	0.754
	*Change*	5.77	2.49			6.00	2.39			5.80	2.45			6.09	2.37		
Peer Px.	*No change*	1.46	1.45	−3.74*	0.257	1.52	1.49	−4.49*	0.312	1.61	1.60	−3.46*	276	1.50	1.47	−3.82*	0.257
	*Change*	1.88	1.76			2.03	1.86			2.06	1.78			1.92	1.79		
Pro social	*No change*	5.99	1.64	−2.32^	0.160	6.10	1.65	−1.17	0.081	6.13	1.61	−0.39	0.031	6.14	1.67	−0.04	0.003
	*Change*	6.25	1.68			6.23	1.70			6.19	1.86			6.15	1.67		
PSS	*No change*	24.40	7.68	−4.82*	0.331	24.85	7.60	−5.47*	0.381	25.11	7.70	−5.90*	0.471	23.77	7.39	−8.48*	0.371
	*Change*	26.99	7.90			27.82	8.10			28.77	7.99			28.11	7.83		
Stressors	*No change*	17.45	6.29	−5.14*	0.353	17.96	6.39	−5.21*	0.636	18.18	6.48	−5.45*	0.435	17.17	6.35	−7.68*	0.517
	*Change*	19.74	6.60			20.31	6.61			21.00	6.44			20.47	6.38		
Rewards	*No change*	6.94	2.82	−1.60	0.110	6.90	2.58	−3.24*	0.225	6.93	2.61	−3.89*	0.310	6.59	2.51	−5.8*	0.391
	*Change*	7.24	2.70			7.51	2.97			7.77	3.09			7.65	2.87		
R-14	*No change*	76.31	11.5	2.48^	0.171	75.64	11.4	1.77	0.123	75.82	11.46	3.34*	0.266	77.11	11.19	5.10	0.343
	*Change*	74.31	11.8			74.20	12.2			72.72	12.32			73.14	11.93		
Competence	*No change*	61.44	8.99	2.16^	0.149	60.92	9.01	1.27	0.088	61.13	9.04	2.99*	0.239	62.07	8.82	4.69*	0.315
	*Change*	60.08	9.30			60.11	9.50			58.94	9.55			59.20	9.35		
Acceptance	*No change*	14.87	3.15	2.89*	0.199	14.72	3.02	2.85*	0.198	14.70	3.09	3.64*	0.291	15.04	3.01	5.2*	0.350
	*Change*	14.23	3.21			14.09	3.44			13.77	3.44			13.94	3.28		

Sx.: symptoms; Px.: problems; *: *p* < 0.001; ^: *p* < 0.05.

**TABLE 5b:** Mean scores, standard deviation and Student t to evaluate the differences in the variables according to the perception of habit change in the children (cont.).

		**Fears**				**Contact with peers**				**Contact with relatives**			
		***M***	***SD***	***t***	***d***	***M***	***SD***	***t***	***d***	***M***	***SD***	***t***	***d***
SDQ	*No change*	11.52	4.74	−11.6*	0.818	11.50	5.71	−4.81*	0.311	12.94	5.59	−0.64	0.02
	*Change*	15.69	5.70			13.51	5.30			13.21	5.09		
Emotional Sx.	*No change*	3.16	1.51	−12.7*	0.899	3.56	1.78	−1.27	0.095	3.70	1.82	0.22	0.130
	*Change*	4.66	1.92			3.74	1.82			3.67	1.80		
Behavioral Px.	*No change*	2.04	1.48	−8.91*	0.627	2.10	1.64	−3.11*	0.168	2.37	1.66	−0.87	0.055
	*Change*	3.04	1.78			2.50	1.66			2.48	1.65		
Hyperactivity	*No change*	4.78	2.45	−6.97*	0.490	4.46	2.72	−5.2*	0.339	5.19	2.60	−0.27	0.117
	*Change*	5.98	2.51			5.46	2.42			5.25	2.31		
Peer Px.	*No change*	1.54	1.53	−4.04*	0.284	1.39	1.48	−3.39*	0.236	1.68	1.67	−1.06	0.202
	*Change*	2.01	1.82			1.82	1.69			1.82	1.58		
Pro social	*No change*	5.99	1.71	−3.87*	0.272	6.44	1.60	3.09*	0.081	6.23	1.63	2.73*	0.450
	*Change*	6.44	1.55			6.05	1.68			5.87	1.77		
PSS	*No change*	25.04	7.80	−4.69*	0.330	24.58	8.25	−3.02*	0.112	25.89	8.02	−0.48	0.137
	*Change*	27.62	7.86			26.42	7.75			26.19	7.58		
Stressors	*No change*	18.07	6.55	−4.67*	0.328	17.62	6.85	−3.22*	0.155	18.88	6.61	0.35	0.025
	*Change*	20.21	6.40			19.25	6.43			18.69	6.45		
Rewards	*No change*	6.97	2.68	−2.31^	0.162	6.97	2.65	−0.98	0.046	7.01	2.67	−2.24^	0.338
	*Change*	7.41	2.84			7.18	2.78			7.50	2.96		
R-14	*No change*	75.99	11.29	3.03*	0.213	78.37	10.60	4.88*	0.286	75.59	11.29	2.23^	0.251
	*Change*	73.50	12.35			74.00	11.90			73.52	12.98		
Competence	*No change*	61.22	8.93	2.63^	0.185	63.21	8.35	4.93*	0.266	61.02	8.79	2.34^	0.271
	*Change*	59.53	9.59			59.74	9.31			59.31	10.32		
Acceptance	*No change*	14.77	3.06	3.57*	0.251	15.16	3.10	3.69*	0.286	14.57	3.14	1.43	0.140
	*Change*	13.97	3.39			14.25	3.20			14.21	3.39		

Sx.: symptoms; Px.: problems; *: *p* < 0.001; ^: *p* < 0.05.

In a linear regression analysis of the total difficulties score (SDQ) as the dependent variable and changes in children’s habits as the independent variable, we found a statistically significant regression model (*F*(4.878) = 88.47; *p* < 0.001), in which the total score on difficulties (*β* = 0.335; *t* = 5.57; *p* < 0.001), behavior problems (*β* = 0.123; *t* = 2.68; *p* < 0.05), emotional symptoms (*β* = 0.093; *t* = 2.35; *p* < 0.005) of the children and resilience competencies of the parents (*β* = –0.097; *t* = –3.304; *p* < 0.005) explained 28% (Adjusted *R*^2^ = 0.284) of variance in changes in habits.

### Mediation Analysis of Resilience as a Mediating Factor Between Parental Stress and Children’s Difficulties

**FIGURE 2:**
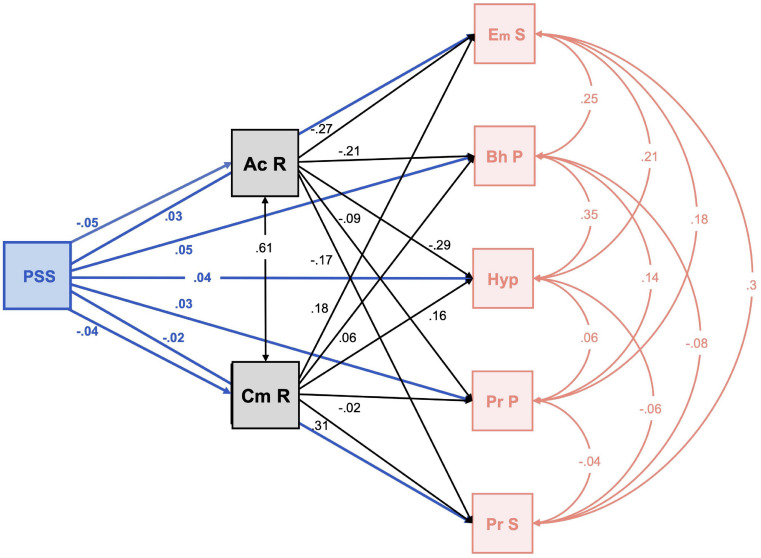
Mediation analysis between parental stress and difficulties in children with resilience as a mediating factor. PPS: parenting stress; Ac R: self-acceptance resilience; Cm P: resilience competences; Em S: emotional symptoms; Bh P: Behavioral problems; Hyp: hyperactivity; Pr P: peer problems; Pr S: prosocial. All lines represents direct effects.

Figure 2 shows the mediation model estimated with the standardized means between directly related variables. This model shows the statistically significant positive direct effects between parental stress and emotional symptoms (*B* = 0.03; *Z* = 6.97; *p* < 001; 95% CI [0.022-0.039]), behavior problems (*B* = 0.05; *Z* = 12.36; *p* < 001; 95% CI [0.042-0.058]), hyperactivity (*B* = 0.04; *Z* = 9.68; *p* < 001; 95% CI [0.032-0.048]) and problems with peers (*B* = 0.028; *Z* = 6.28; *p* < 001; 95% CI [0.019-0.036]), and negative for prosocial behavior (*B* = −0.17; *Z* = −3.85; *p* < 001; 95% CI[−0.026–0.008]).

With acceptance of oneself as a meditating factor in parental stress, there were statistically significant positive indirect effects on emotional symptoms (*B* = 0.012; *Z* = 5.1; *p* < 001; 95% CI [0.008-0.017]), behavior problems (*B* = 0.01; *Z* = 4.43; *p* < 001; 95% CI [0.005-0.014]), hyperactivity (*B* = 0.013; *Z* = 5.64; *p* < 001; 95% CI [0.009-0.018]) and prosocial behavior (*B* = 0.008; *Z* = 3.46; *p* < 001; 95% CI [0.003-0.012]). However, with personal competence in resilience as a mediator of stress, there were statistically significant negative indirect effects on emotional symptoms (*B* = −0.008*Z* = −3.58; *p* < 001; 95% CI [-0.012-0.017]), hyperactivity (*B* = −0.007*Z* = −3.55; *p* < 001; 95% CI [−0.011- −0.003]) and prosocial behavior (*B* = −0.013; *Z* = −5.53; *p* < 001; 95% CI [0.018-0.008]).

## Discussion

At the beginning of the pandemic, there was not yet clear evidence of the cost/benefits of social distancing measures, and specifically, school closures for controlling contagion by COVID-19. However, the urgency of identifying its impact on the children-youth population, and how the routines of parents and children could safely be returned to (especially where family conciliation strategies were needed) due to the economic and psychosocial consequences that this distancing could have on both, was already apparent (Viner et al., 2020). In the first months of the year, the lack of research on this topic was also clear (Orben et al., 2020; Viner et al., 2020), although there was agreement in emphasizing the severity of consequences, especially among the more vulnerable groups, such as the children-youth population (Brooks et al., 2020; Espada et al., 2020; Fore, 2020; Lee, 2020; Liu J. J. et al., 2020; Rosenthal et al., 2020; Viner et al., 2020), noting alteration in routines and stress as two of the factors that most affect physical and psychological wellbeing in a situation of lockdown and social distancing (Balluerka et al., 2020; Wang et al., 2020). Currently, several studies have revealed the negative emotional impact of home confinement in different continents (Ammar et al., 2020b). As mentioned by García Ron and Cuéllar-Flores (2020), although there are expert recommendations for mitigating the negative effects of quarantine, there is still little scientific evidence on the protective factors against the psychological impact that confinement could have on children and adolescents. Therefore, the present study was focused on describing and analyzing the relationship between the difficulties in psychological adjustment and the change of habits of the infant-juvenile population as perceived by their parents and their stress and resilience, as well as to analyze the course of the changes and the relationships between the third and sixth weeks of confinement of the first wave of the COVID-19 in Spain.

Regarding the descriptive results obtained to meet the first objective of this study, it is globally confirmed that parents perceive a change in the habits of the infant-juvenile population. In particular, these results confirmed a change in the habits of the children-youth population, especially those related to difficulties in contact with peers and family members and sleep problems, coinciding with other studies (Berasategui Sancho et al., 2020; Brooks et al., 2020; Lee, 2020; Orgilés et al., 2020; Serrano-Martínez, 2020). As reviewed by Cifuentes-Faura (2020) school closures can lead to consequences, especially among the most vulnerable, related to eating insecurity or less healthy diets, less physical activity, and more screen use, with negative effects to their mental and physical health, made worse by interaction between changes in lifestyle and psychosocial stress caused by lockdown. Also, a multi-center multi-country study in adults by Ammar et al. (2020a) revealed that physical activity has decreased and sitting hours have increased.

Along with the modification of routines and habits, the results show that parents also perceive psychological difficulties in their children, with cutoff scores showing their problematic susceptibility in emotional symptomatology, behavior problems, hyperactivity, prosocial behavior, and total difficulties, except problems with peers where mean scores were in the ranges of normality. As noted by Quezada-Scholz (2020), the threat COVID-19 poses in different facets of life can generate uncertainty and fear, which in turn, would accelerate disproportionate avoidance and even aggressive behaviors that could explain this perception of behavior difficulties in general and directly related to behavior problems, hyperactivity, and prosocial behavior difficulties.

Some studies have identified emotional symptomatology and symptomatology related it to parental perception of hyperactivity (Orgilés et al., 2020; Tiwari et al., 2020). Over 70% of parents interviewed during lockdown perceived that their children were more nervous and got angry more easily, and over 50% stated that their children cried more than before and were sadder (Berasategui Sancho et al., 2020). Other studies (Serrano-Martínez, 2020) beyond this symptomatology, emphasize emotions of joy, and Berasategui Sancho et al. (2020) underlined emotional ambivalence in childhood. In adults, Balluerka et al. (2020) emphasized the changes in dysphoric mood states (resignation, sadness, uncertainty and fear, and others) and to a lesser extent, also euphoric (the positive side of family life, less stress, normalization of emotions over time, and others) although referring to prosocial conduct, they highlight behaviors, such as applause for healthcare workers and helping neighbors, and in general, the perception of solidarity and social cohesion.

In the study on parental stress, we confirmed that, although scores were above the mean, families showed moderate stress, with significant differences regarding children’s ages, decreasing as the children were older and finding that parents of the smallest children were those who reported the most problems adjusting to this environment. Among the tools and resources recommended for minimizing the effects of lockdown and favoring better social and family coping are the use of information technologies and social communication, as they can supplement the lack of social contact. However, their use is more complicated for the smaller children (Bazán et al., 2020; Brooks et al., 2020; Orben et al., 2020).

At the same time, our results describe parents with a high level of resilience, which is evident in Personal Competence scores above the mean and Acceptance of oneself and of life, slightly below. Characterizing resilience then as a positive personality trait, the families studied showed sufficient capacity to adapt to the adversities arising from lockdown (Wagnild and Young, 1993), where skills developing resilience had more weight than the more intrinsic matters that facilitate its development (Gómez, 2019). Although depending on the type of problem, resilience can vary (Cantero-García and Alonso-Tapia, 2018), these results agree with those found in sports (Trigueros et al., 2017) and with respect to total resilience in a sample of parents of children with disabilities, and in a sample of adults with a motor disability (Suriá Martínez, 2013, 2014, among others).

In view of these results, we can confirm, with respect to our first hypothesis, the presence of psychological adjustment difficulties of parents, children, and adolescents. However, of more interest than the snapshot is the study of changes which occurred as lockdown continued.

Thus, we found that the longer lockdown lasted, the stronger the perception of change in habits, an aspect which has been emphasized in other studies in adult populations (Levy et al., 2020; Ammar et al., 2021), which have shown changes in eating habits, physical exercise, sleep and hygiene, among others, during lockdown. Also, in agreement with previous studies (Brooks et al., 2020; Gómez-Becerra et al., 2020; Sandín et al., 2020), it was confirmed that the duration of lockdown accentuated parents’ perception of the psychological difficulties of their children, supporting the second hypothesis. As Gómez-Becerra et al. (2020) mentioned, the symptomatology observed in COVID-19 studies before or at the beginning of lockdown is less than at later times. In this study, only prosocial conduct of children remained stable throughout lockdown, with susceptibility to becoming problematic during the entire period, and finding variability between weeks in problems with peers. As mentioned above, the uncertainty and fear caused by COVID-19 could cause avoidance and aggressive behaviors (Quezada-Scholz, 2020), which along with the lack of social contact during lockdown could explain these results.

We also observed a decrease in resilience, more in the Acceptance of oneself and of life component than in the Personal competence component. That is, lockdown affected acceptance of circumstances and a feeling of peace more than the characteristic skills of the resilient person (Wagnild and Young, 1993), despite experiencing adversity. This could be explained to the extent that perceived stress and its consequent inverse relationship with resilience has a greater effect on the Acceptance component than Competence, probably due to the anomalous situation. As Villaceiros Durbán (2017) demonstrated in her review of studies on resilience, the effect of duration of adversity has contradictory results, and our study is aligned with those that have found a decrease in resilience with persistence of the negative circumstance of life. In view of the above, we can confirm our second hypothesis that psychological adjustment problems would increase with the duration of lockdown.

Moreover, the results reveal that parents perceive different problems depending on the ages of their children, finding more change in habits and more behavior problems and hyperactivity in those under three years old; more emotional and prosocial symptoms from six to twelve and more problems with peers in girls and boys in middle school (12 to 16).

Although there are no studies for comparing our results on the resilience of parents by their children’s grade level, the fact that parents showed more resilience as children were older is congruent with those found by other researchers, as in our study (Becoña et al., 2013; Sánchez-Teruel and Robles-Bello, 2015), the age of parents increased as children passed from one grade to another, thereby supporting the idea that resilience is not a static trait, but rather a dynamic process modifiable throughout the life cycle (Villalba Quesada, 2003; Windle, 2011; García Renedo et al., 2013; Sánchez-Teruel and Robles-Bello, 2014; Fernandes de Araújo et al., 2015; Jiménez Pina, 2016; García-León et al., 2019). Furthermore, the presence of higher levels of self-reported resilience of parents with older children (middle and high school) could show, on one hand, the effect of cumulative parental experience, and on the other, the decreasing need for social support (strongly linked to Resilience; e.g., Villaceiros Durbán, 2017; Prime et al., 2020) for caregiving and employment conciliation in bringing up children. However, we should mention that other studies have shown the opposite (Saavedra Guajardo and Villalta Paucar, 2008), in the sense that younger adults are those who have higher scores in resilience then older adults, and even some that explicitly mention the inexistence of significant relationships between demographic variables such as age, for instance, and resilience (Zarzaur et al., 2017).

Regarding the second objective of the study, that is, to analyze the differences in the perception of psychological difficulties and change of habits of the children, according to the age of the children and/or the week of lockdown, we can highlight the following points:

When, in addition, the effects of duration of lockdown and age of children were combined, significant effects were found in parental perception of hyperactivity of children from three to six years. As mentioned by Echeburúa et al. (2004), and reported by Bazán et al. (2020), change in habits and certain emotional symptoms may be a product of lack of skills for understanding the situation and expressing emotions in smaller children. In the same line, Espada et al. (2020), citing the National Child Traumatic Stress Network, mentioned that nightmares, fear, aggressive behavior, change in appetite and more tantrums, complaints or attachment behaviors could be expectable behavior in children under six years old. Irritability, sleep or appetite problems, physical symptoms, behavior problems, excessive attachment, competition for attention of parents and loss of interest in friends, may be common from six to twelve, and from 13 to 18, physical symptoms, sleep and appetite problems, isolation from friends and loved ones, as well as changes in energy, apathy and loss of interest in health behaviors. As emphasized by Orben et al. (2020), adolescence is a sensitive period of development in which the lack of interaction among peers could have short and long-term effects on social, cognitive and behavioral development, although they also reported the need for more research in this area. As described above, our study results showed difficulties in prosocial behavior and also with regard to changes in fears and contact with peers and family members during lockdown. Considering the information compiled by Espada et al. (2020), the confinement situation due to the pandemic leads to changes in fears and lack of social contact, promotes loss of interest in friends and isolation from them and from loved ones, which could explain the difficulties perceived in prosocial behavior.

In this sense, we can also confirm our third hypothesis related to the influence of children’s ages and grade level on parents’ perception of their psychological difficulties and changes in habits.

The analysis of family variables confirmed a two-way relationship, in relation to the third objective of the study. The correspondence between change in habits and behavior difficulties showed a two-way relationship between the two, except for the relationship between perception of change in habits and prosocial behavior, where no significant differences were found. Probably, the parental perception of their children’s difficulties made it harder to keep up certain routines. Thus, a change in routines could lead to bad temper, frustration and anxiety (Lee, 2020). Specifically, previous studies (León Martínez et al., 2020) have found susceptibility to depressive, anxiety, and eating disorders and to screen addiction, one of the most frequent changes in habits in our study.

This change in habits of children and adolescents, in addition to the behavior problems in this population, are related to parental stress. Thus, management of behavior children’s problems (aggressiveness, rebelliousness, social and emotional problems, disobedience) can become a stressful life situation that can increase parental stress as it decreases resilience (Cantero-García and Alonso-Tapia, 2018). The role of parental stress in childhood development and behavior, affects the perception parents have of their children, and this, the quality of couple interaction (Crnic and Low, 2002; Mackintosh et al., 2006; Mitchell and Hauser-Cram, 2010; Fernández-Rodríguez et al., 2015). Mash and Johnston (1990), in their description of the three domains (parents, children and interaction), stated that the presence of stress in any one of them negatively influences the remaining two. Thus, we confirmed, as expected in this context, higher scores in parental stress, difficulties in interaction as mentioned above, such as use of more coercive strategies (Burke et al., 2008; Respler-Herman et al., 2011), more dysfunctional practices, and lower levels of affect and communication (Bonds et al., 2002), as well as children’s internalization and externalization problems (Costa et al., 2006). This confirms the fourth hypothesis.

There was a statistically significant negative relationship between the change in habits in children-youth population and parental resilience, suggesting that as parental perception of changes in habits increased, their resilience lessened. In fact, the most resilient parents were those who perceived fewer changes in their children’s habits than less resilient parents who reported greater changes in children’s routines. In line with these results, resilience is argued as a facilitating element for maintaining family habits and routines during COVID-19 (Prime et al., 2020).

A statistically significant inverse relationship between stress and resilience shown in other studies was also confirmed (hypothesis 5). Focusing on general stress and not a clinical sample, García-León et al. (2019) identified a significant inverse/negative correlation between resilience and depression, anxiety, vulnerability to stress and perceived stress. The same is true of single mothers of adolescents, vulnerable adolescents, mothers with newborn babies, women victims of trauma, and older women, underlining the inverse association between resilience and stress, depression, anxiety, loneliness and desperation (see a review by Wagnild, 2009). This relationship between resilience and stress has also been found in caregivers of patients in hemodialysis (Martínez-Rodríguez et al., 2019). Focusing on parental stress, and derived from studies with a clinical sample, that is parents of children with neurodevelopmental disorders (Cara García, 2019), parents of children with ASD (Jiménez Pina, 2016; Tijeras Iborra, 2017); parents of hyperactive, autistic, intellectually disabled children or who have learning difficulties (Suzuki et al., 2018), physically, intellectually disabled or pluri-disabled and ASD children (Vela Llauradó and Suárez Riveiro, 2020), also back the negative relationship between resilience and stress. As noted by Sánchez-Teruel and Robles-Bello (2014), there are few studies on resilience in non-clinical populations exposed to constant adverse and traumatic situations. And although confinement and social distancing derived from the pandemic have not been nor permanent measures, COVID-19 is beginning to be a constant threat in our lives and a source of stress for the population. There is much evidence on the protective role of resilience against perceived stress (Fernandes de Araújo et al., 2015; García-León et al., 2019), as well as that psychological resources, such as self-esteem, internal control, or coping through emotional search mitigate and can even eliminate the adverse effect of stress and favor resilience. We can therefore confirm the hypothesis asserting that families with higher resilience show less parental stress.

The fourth objective of this study was to evaluate the influence of changes in habits on parental factors and children’s problems. The regression analysis performed revealed an explanatory model of change in habits depending on behavior problems, emotional symptoms and total psychological problems of the children, as well as parents’ resilience. The results show that parents who report changes in their children’s habits perceive more psychological difficulties in their children and more self-perceived stress than parents who do not perceive these changes in their children’s habits, thus confirming hypothesis 6. Thus, keeping or changing routines, understood as managing the family situation, depends on the responses to change and emotional symptoms, spaces for play and lack of social contact, which also vary depending on the level of the child’s maturity and development during lockdown, as mentioned by Merino-Navarro and Díaz-Periánez (2020), and the parents’ capacity to confront adversity, in this case lockdown, all become predictive factors of the perception of change in children-youth habits.

The fifth objective was to analyze resilience as a mediating factor between parental stress and childhood problems during lockdown. In the mediation analysis, we saw how parental stress had a direct positive effect on children’s difficulties. This result has been observed previously in aggressive behavior of adolescents (Cabrera et al., 2012), behavior, social and emotional problems of children with ADHD (Theule et al., 2013; Berenguer et al., 2019), in children with ASD (Romero-González et al., 2020), and others. However, when we included the resilience variable in the analysis, this effect continued in the same direction as acceptance of oneself and of life. That is, when adaptability, balance, flexibility and a stable perspective of life (Factor II of ER-14) acted as a mediator. But the relationship was the opposite with stress, when parents showed personal resilience competencies, that is, self-confidence, independence, decision, ingenuity and perseverance (Factor I of ER-14). Thus, we can suggest that parental skills of resilience and personal competence act as a protective factor or mediator in their children’s problems, since with acceptance of oneself and of life, the relationship between parental stress and psychological problems of the children is weakened, and with personal competence in resilience, the relationship is reversed, as it acts as a mediating element in this relationship, becoming a protective factor against the perception of the psychological problems of their children. Therefore, we accept Hypothesis 7, in which resilience of parents mediated the relationship between parental stress and psychological problems of their children. In this line, and from a psychosocial approach in the area of migration, Villaceiros Durbán (2017) analyzed the behavior of resilience as a buffer between migratory stress and psychological health of adolescent migrants, confirming the protective and moderating effect of resilience (family strengths and affective support).

## Limitations of the Present Study

One limitation of our study is that, although we found whether there was a change in routines related to social contact, sleep or eating, and others, we do not know the direction of the change. The study by Wang et al. (2020) at the beginning of lockdown in China, found that the change in certain habits (specifically, hygiene, such as not using chopsticks at meals or washing hands) was associated with lower scores on the psychological impact scales and stress, depression and anxiety symptoms in those surveyed over 21 years of age. Another limitation and future directions would be the fact that, as in most research studies in the family setting, most of the participants are women. Finally, it would have been relevant to carry out a longitudinal follow-up of the families participating in the study. The research methodology used for collecting data, as well as the ethical considerations regarding the anonymity of the families did not make it possible. To solve this problem, the data were compared differentially according to the week of the confinement period (i.e., whether it was the third or the sixth week) in which the surveys were carried out.

## Conclusion and Practical Implications

The conclusions that can be derived from this study suggest that lockdown caused by COVID-19 has had a considerable effect on families, both on children and parents, which has been reflected in higher levels of stress and different types of difficulties. This effect has grown as the duration of lockdown has continued, and so we celebrate government decisions around the world to moderate confinement for children. What has been suggested as an economic requirement is also an emotional one. The fact that all families have not coped with this situation with the same efficacy, since those who have been able to provide a stable setting managing routines of activities, leisure, hygiene and sleep had lower scores in stress and childhood difficulties provides us with a guide for intervention as psychology and education professionals.

The results obtained suggest that the guidelines that should be indicated to families would be in the following direction:

(1) Parents should practice or learn emotional regulation strategies that allow them to reduce their parental stress and organize their families in order to reduce stress and overburden in childcare and parenting.
(2) They should practice their resilience skills, either in their strengths, already applied in other adverse situations. They should either work on their resilience under the guidance of psychologists to improve their coping strategies, their acceptance skills, their tolerance to discomfort and frustration, and their capacity for psychological flexibility (cognitive and behavioral).
(3) Parents should organize their children’s schedules, routines and activities in a confinement period, by applying variability, rest times, avoiding saturation or overdoses of any activity (neither academic, nor video games, nor television, etc.), being creative in the development of innovative activities for their children, such as the use of information technologies to supply the lack of social contact, special systematization in playing with them and sharing common interests.
(4) Finally, parents should be careful about the type of information provided to their children about the pandemic situation, avoiding negative, bleak, catastrophic, or fear-based information. On the contrary, parents should encourage intelligent optimism (e.g., even though in this confinement they cannot do what they would like to do, it may be an opportunity to learn new activities, to share more time with parents, etc.). On the other hand, parents should raise their children’s awareness of their own

resilience, encourage their attitudes of solidarity and gratitude, etc.

## Data Availability Statement

The raw data supporting the conclusions of this article will be made available by the authors, without undue reservation.

## Ethics Statement

The studies involving human participants were reviewed and approved by Bioethic Committee of University of Almería (Spain). The patients/participants provided their written informed consent to participate in this study.

## Author Contributions

PS-L was involved in the management of the research project and the ethical permissions. IG-B worked on the development and supervision of the design of methodology. JF-C was involved in data curation and formal analysis. MA-R and MF-T wrote the original draft introduction and conclusions of the study. All authors were involved in the design of the study, writing of the final review and editing, and contributed equally to this study.

## Conflict of Interest

The authors declare that the research was conducted in the absence of any commercial or financial relationships that could be construed as a potential conflict of interest.

## Appendix 1.

### Self-Report Habits *of Everyday Living*

Here are a few questions about your children’s everyday habits and how much you think they changed during lockdown?

Please select only one of the following options:

(1) Not at all, (2) Not much, (3) Neutral, (4) Somewhat (5) Very much
1. To what extent have you noticed in the last two weeks if your child had difficulty to maintain his/her **sleep routines?**
2. To what extent have you noticed in the last two weeks if your child had difficulty to maintain his/her **eating habits?**
3. To what extent have you noticed in the last two weeks if your child had difficulty to maintain his/her **hygiene habits?**
4. To what extent have you noticed in the last two weeks if your child had difficulty to maintain a **stable mood?**
5. To what extent have you noticed in the last two weeks if your child has **felt afraid?**
6. To what extent have you noticed in the last two weeks whether your child has **kept in contact with friends and/or classmates?**
7. To what extent have you noticed in the last two weeks if your child **has kept in contact with family members who are not at home?**

## References

[B1] Ammar A. Brach M. Trabelsi K. Chtourou H. Boukhris O. Masmoudi L. (2020a). Effects of COVID-19 home confinement on eating behaviour and physical activity: results of the ECLB-COVID19 international online survey. *Nutrients* 12:1583. 10.3390/nu12061583 32481594 PMC7352706

[B2] Ammar A. Mueller P. Trabelsi K. Chtourou H. Boukhris O. Masmoudi L. (2020b). Psychological consequences of COVID-19 home confinement: the ECLB-COVID19 multicenter study. *PLoS One* 15:e0240204. 10.1371/journal.pone.0240204 33152030 PMC7643949

[B3] Ammar A. Trabelsi K. Brach M. Chtourou H. Boukhris O. Masmoudi L. (2021). Effects of home confinement on mental health and lifestyle behaviours during the COVID-19 outbreak: insights from the ECLB-COVID19 multicentre study. *Biol. Sport* 38:9. 33795912 10.5114/biolsport.2020.96857 PMC7996377

[B4] Anastopoulos A. D. Guevremont D. C. Shelton T. L. DuPaul G. J. (1992). Parenting stress among families of children with attention deficit hyperactivity disorder. *J. Abnorm. Child Psychol.* 20 503–520. 10.1007/BF00916812 1487593

[B5] Ayala-Nunes L. Lemos I. Nunes C. (2014). Predictores del estrés parental en madres de familia en riesgo psicosocial. *Univ. Psychol.* 13 529–539. 10.11144/Javeriana.UPSY13-2.pepm

[B6] Balluerka N. Gómez J. Hidalgo M. D. Gorostiaga A. Espada J. P. Padilla J. L. (2020). *Las Consecuencias Psicológicas de la COVID-19 y el confinamiento. Informe de investigación.* Biscay: Universidad del País Vasco.

[B7] Bazán C. Brückner F. Giacomazzo D. Gutiérrez M. A. Maffeo F. (2020). *Adolescentes, COVID-19 y Aislamiento Social, Preventivo y Obligatorio.* New York, NY: FUSA AC.

[B8] Becoña E. (2006). Resiliencia: definición, características y utilidad del concepto. *Rev. Psicopatol. Psicol. Clín.* 11 125–146. 10.5944/rppc.vol.11.num.3.2006.4024

[B9] Becoña E. López-Durán A. Fernández del Río E. (2013). Resiliencia y consumo de cannabis, drogas de síntesis y cocaína en jóvenes. *Psicopatol. Clín. Legal Forense* 13 59–72.

[B10] Berasategui Sancho N. Idoiaga Mondragon N. Dosil Santamaría M. Picaza Gorrochategui M. Ozamiz Etxebarria N. (2020). *Las Voces de Los Niños y De Las Niñas en Situación de Confinamiento Por el COVID-19.* País Vasco: Universidad de País Vasco.

[B11] Berenguer C. Roselló B. Baixauli I. (2019). Perfiles de familias con factores de riesgo y problemas comportamentales en niños con déficit de atención con hiperactividad. *Int. J. Dev. Educ. Psychol.* 2 75–84.

[B12] Berry J. O. Jones W. H. (1995). The parental stress scale: initial psychometric evidence. *J. Soc. Pers. Relationsh.* 12 463–472. 10.1177/0265407595123009

[B13] Bitsika V. Sharpley C. F. Bell R. (2013). The buffering effect of resilience upon stress, anxiety, and depression in parents of a child with an autism spectrum disorder. *J. Dev. Phys. Disabil.* 25 533–543. 10.1007/s10882-013-9333-5

[B14] Bonds D. D. Gondoli D. M. Sturge-Apple M. L. Salem L. N. (2002). Parenting stress as a mediator of the relation between parenting support and optimal parenting. *Sci. Pract.* 2 409–435. 10.1207/S15327922PAR0204_04

[B15] Brooks S. K. Webster R. K. Smith L. E. Woodland L. Wessley S. Greenberg N. (2020). The psychological impact of quarantine and how to reduce it: rapid review of evidence. *Lancet* 395 912–920. 10.1016/S0140-6736(20)30460-8 32112714 PMC7158942

[B16] Burke J. D. Pardini D. A. Loeber R. (2008). Reciprocal relationships between parenting behavior, and disruptive psychopathology from childhood through adolescence. *J. Abnorm. Child Psychol.* 36 679–692. 10.1007/s10802-008-9219-7 18286366 PMC2976977

[B17] Byrne E. A. Cunningham C. C. (1985). The effects of mentally handicapped children on families. A conceptual review. *J. Child Psychol. Psychiatry* 26 847–864. 10.1111/j.1469-7610.1985.tb00602.x 2933420

[B18] Cabrera V. E. González M. R. Guevara I. P. (2012). Estrés parental, trato rudo y monitoreo como factores asociados a la conducta agresiva. *Univ. Psychol.* 11 241–254. 10.11144/javeriana.upsy11-1.eptr

[B19] Cameron S. J. Dobson L. A. Day D. M. (1991). Stress in parents of developmentally delayed and non-delayed preschool children. *Canada’s Mental Health* 39 13–17.

[B20] Cantero-García M. Alonso-Tapia J. (2018). Escala breve de resiliencia frente a los problemas de comportamiento de los hijos (EBR-PC). *Anales de Psicología* 34 531–535. 10.6018/analesps.34.3.312601

[B21] Cara García R. (2019). *Estrés Parental y Resiliencia en Padres de Hijos Con Trastornos del Neurodesarrollo.* Trabajo Fin de Master. La Cañada: Universidad de Almería.

[B22] Ceballo R. McLoyd V. C. (2002). Social support and parenting in poor, dangerous neighborhoods. *Child Dev.* 73 1310–1321. 10.1111/1467-8624.00473 12146749

[B23] Chaudry A. Wimer C. (2016). Poverty is not just an indicator: the relationship between income, poverty, and child well-being. *Acad. Pediatr.* 16 S23–S29. 10.1016/j.acap.2015.12.010 27044698

[B24] Cifuentes-Faura J. (2020). Consecuencias en los niños del cierre de escuelas por COVID-19: el papel del gobierno, profesores y padres. *Rev. Int. Educ. Para Just. Soc.* 9:12216.

[B25] Costa N. M. Weems C. F. Pellerin K. Dalton R. (2006). Parenting stress and childhood psychopathology: an examination of specificity to internalizing and externalizing symptoms. *J. Psychopathol. Behav. Assess.* 28 113–122. 10.1007/s10862-006-7489-3

[B26] Crnic K. Low C. (2002). “Everyday stresses and parenting,” in *Handbook of Parenting: Practical Issues in Parenting*, ed. Bornstein M. H. (Mahwah, NJ: Lawrence Erlbaum Associates Publishers), 243–267.

[B27] Cronin S. Becher E. H. (2015). “Research summary,” in *Parents and Stress: Understanding Experiences, Context and Responses: Children’s Mental Health eReview*, eds Cronin S. Becher E. H. Maher K. S. M. Debb S. (St. Paul, MN: University of Minnesota Extension, Children, Youth and Family Consortium).

[B28] Deater-Deckard K. (2004). *Parenting Stress.* New Haven, CT: Yale University Press.

[B29] Díaz-Herrero A. Brito de la Nuez A. G. López Pina J. A. Pérez-López J. Martínez-Fuentes M. T. (2010). Estructura factorial y consistencia interna de la versión española del parenting stress index-short form. *Psicothema* 22 1033–1038. 21044549

[B30] Duncan G. J. Brooks-Gunn J. (2000). Family poverty, welfare reform, and child development. *Child Dev.* 71 188–196. 10.1111/1467-8624.00133 10836573

[B31] Echeburúa E. Corral P. D. Amor P. J. (2004). Nuevos enfoques terapéuticos del estrés postraumático en víctimas de terrorismo. *Clín. Salud* 15 273–292.

[B32] Espada J. P. Orgilés M. Piqueras J. A. Morales A. (2020). Las buenas prácticas en la atención psicológica infanto-juvenil ante el COVID-19. *Clín. Salud* 31 109–113. 10.5093/clysa2020a14

[B33] Español-Martín G. Pagerols M. Prat R. Rivas C. Sixto L. Valero S. (2020). Strengths and difficulties questionnaire: psychometric properties and normative data for Spanish 5-to 17-Year-olds. *Assessment* [Epub ahead of print]. 10.1177/1073191120918929 32449368

[B34] Estes A. Munson J. Dawson G. Koehler E. Zhou X. Abbott R. (2009). Parenting stress and psychological functioning among mothers of preschool children with autism and developmental delay. *Autism* 13 375–387. 10.1177/1362361309105658 19535467 PMC2965631

[B35] Farmer A. Y. Lee S. K. (2011). The effects of parenting stress, perceived mastery, and maternal depression on parent–child interaction. *J. Soc. Serv. Res.* 37 516–525. 10.1080/01488376.2011.607367

[B36] Fernandes de Araújo L. Teva I. Bermúdez M. P. (2015). Resiliencia en Adultos: una revisión teórica. *Ter. Psicol.* 33 257–276. 10.4067/s0718-48082015000300009 27315006

[B37] Fernández-Rodríguez L. Rodríguez-Sarmiento A. Armada-Gordo E. (2015). ¿Cómo se enfrentan los padres al estrés que se genera ante la discapacidad de un hijo? *Rev. Estudios Investig. Psicol. Educ.* 05 019–023. 10.17979/reipe.2015.0.05.146

[B38] Folkman S. Lazarus R. S. (1985). If it changes it must be a process: study of emotion and coping during three stages of a college examination. *J. Pers. Soc. Psychol.* 48 150–170. 10.1037/0022-3514.48.1.150 2980281

[B39] Fore H. (2020). A wake-up call: COVID-19 and its impact on childrens’s health and wellbeing. *Lancet* 8:901. 10.1016/S2214-109X(20)30238-2 PMC7217644 32405458

[B40] García del Castillo J. A. García del Castillo-López A. López-Sánchez C. Dias P. (2016). Conceptualización teórica de la resiliencia psicosocial y su relación con la salud. *Salud Drogas* 16 59–68. 10.21134/haaj.v16i1.263

[B41] García Renedo M. Mateu Pérez R. Flores Buils R. Gil Beltrán J. M. (2013). La resiliencia y las víctimas de desastres. *Cuadernos Crisis Emerg.* 12 1–12.

[B42] García Ron A. Cuéllar-Flores I. (2020). Impacto psicológico del confinamiento y cómo mitigar sus efectos: revisión rápida de la evidencia. *Anales Pediatr.* 93 57–58. 10.1016/j.anpedi.2020.04.015 32553732 PMC7174150

[B43] García-León M. A. González-Gómez A. Robles-Ortega H. Padilla J. L. Peralta-Ramírez M. I. (2019). Propiedades psicométricas de la Escala de Resiliencia de Connor y Davidson (CD-RISC) en población española. *Anales Psicol.* 35 33–40.

[B44] Giallonardo V. Sampogna G. Del Vecchio V. Luciano M. Albert U. Carmassi C. (2020). The impact of quarantine and physical distancing following COVID-19 on mental health: study protocol of a multicentric italian population trial. *Front. Psychiatry* 11:533. 10.3389/fpsyt.2020.00533 32581895 PMC7290062

[B45] Gómez M. A. (2019). *Estandarización de la Escala de Resiliencia de Wagnild & Young en Universitarios de Lima Metropolitana.* Tesis de Licenciatura. Santiago de Surco: Repositorio Institucional Universidad Ricardo Palma.

[B46] Gómez-Becerra I. Flujas J. M. Andrés M. Sánchez-López P. Fernández-Torres M. (2020). Evolución del estado psicológico y el miedo en la infancia y adolescencia durante el confinamiento por la COVID-19. *Rev. Psicol. Clín. Niños Adolescentes* 7 11–18.

[B47] Goodman R. (1997). The strengths and difficulties questionnaire: a research note. *J. Child Psychol. Psychiatry* 38 581–586. 10.1111/j.1469-7610.1997.tb01545.x 9255702

[B48] Gupta V. B. (2007). Comparison of parenting stress in different developmental disabilities. *J. Dev. Phys. Disabil.* 19 417–425. 10.1007/s10882-007-9060-x

[B49] Hayes S. A. Watson S. L. (2013). The impact of parenting stress: a meta-analysis of studies comparing the experience of parenting stress in parents of children with and without autism spectrum disorder. *J. Autism. Dev. Disord.* 43 629–642. 10.1007/s10803-012-1604-y 22790429

[B50] Hewitt Ramírez N. Juárez F. Parada Baños A. J. Romero Chávez Y. M. Vargas Amaya M. V. (2016). Afectaciones psicológicas, estrategias de afrontamiento y niveles de resiliencia de adultos expuesto al conflicto armado en Colombia. *Rev. Colomb. Psicol.* 25 125–140. 10.15446/rcp.v25n1.49966

[B51] Holly L. E. Fenley A. R. Kritikos T. K. Merson R. A. Abidin R. R. Langer D. A. (2019). Evidence-base update for parenting stress measures in clinical samples. *J. Clin. Child Adolesc. Psychol.* 48 685–705. 10.1080/15374416.2019.1639515 31393178

[B52] Imai N. Gaythorpe K. A. M. Abbot S. Bhatia S. van Elsland S. Prem K. (2020). Adoption and impact of non-pharmaceutical interventions for COVID-19. *Welcome Open Res.* 5:59. 10.12688/wellcomeopenres.15808.1 32529040 PMC7255913

[B53] Ispa J. M. Fine M. A. Halgunseth L. C. Harper S. Robinson J. Boyce L. (2004). Maternal intrusiveness, maternal warmth, and mother–toddler relationship outcomes: variations across low-income ethnic and Acculturation Groups. *Child Dev.* 75 1613–1631. 10.1111/j.1467-8624.2004.00806.x 15566369

[B54] Jiménez Pina E. (2016). *Resiliencia en Padres y Madres de Niños con Trastornos del Espectro Autista.* Tesis Doctoral. València: Universidad de Valencia.

[B55] Kim D. (2017). Relationship between caregiving stress depression, and self-steem in family caregivers of adults with disability. *Occup. Ther. Int.* 2017:1686143. 29114184 10.1155/2017/1686143 PMC5664279

[B56] Lazarus R. S. Folkman S. (1986). “Cognitive theories of stress and the issue of circularity, dynamic of stress, 63-80,” in *Dynamics of Stress. The Plenum Series on Stress and Coping*, eds Appley M. H. Trumbull R. (Boston, MA: Springer).

[B57] Lee J. (2020). Mental health effects of school closures during COVID-19. *Lancet Child Adolesc. Health* 4:421. 10.1016/S2352-4642(20)30109-7 32302537 PMC7156240

[B58] León Martínez C. P. Cancino Fernández M. González Cid A. Mesa Latorre T. (2020). Hábitos y trastornos del sueño en población pediátrica: un problema de salud pública. *Rev. Chilena Psiquiatr. Neurol. Infancia Adolesc.* 31 21–28.

[B59] Levy T. S. Gómez L. M. Mundo V. Cuevas L. Gaona E. B. Ávila M. A. (2020). *ENSARS-COV-2: Resultados de la Evaluación Basal de la Encuesta Nacional de las Características de la Población Durante la Pandemia de COVI-19.* Cuernavaca: Instituto Nacional de Salud Pública de México.

[B60] Liu J. J. Bao Y. Huang X. Shi J. Lu L. (2020). Mental health considerations for children quarantined because of COVID-19. *Lancet Child Adolesc. Health* 4 347–349. 10.1016/S2352-4642(20)30096-1 32224303 PMC7118598

[B61] Liu N. Zhang F. Wei C. Jia Y. Shang Z. Sun L. (2020). Prevalence and predictors of PTSS during COVID-19 outbreak in China hardest-hit areas: gender differences matter. *Psychiatry Res.* 287:112921. 10.1016/j.psychres.2020.112921 32240896 PMC7102622

[B62] Lozano-Díaz A. Fernández-Prados J. S. Figueredo Canosa V. Martínez Martínez A. M. (2020). Impactos del confinamiento por el COVID-19 entre universitarios: satisfacción vital, resiliencia, y capital social online. *Int. J. Sociol. Educ.* 2020 79–104. 10.17583/rise.2020.5925

[B63] Mackintosh V. H. Myers B. J. Kennon S. S. (2006). Children of incarcerated mothers and their caregivers: factors affecting the quelity of their relationship. *J. Child Fam. Stud.* 15 581–596. 10.1007/s10826-006-9030-4

[B64] Martínez-Rodríguez L. Fernández-Castillo E. González-Martínez E. Vázquez-Morales H. L. (2019). Apoyo social y resiliencia: factores protectores en cuidadores principales de pacientes en hemodiálisis. *Enfermería Nefrol.* 22 130–139. 10.4321/S2254-28842019000200004

[B65] Mash E. J. Johnston C. (1990). Determinants of parenting stress: illustrations from families of hyperactive children and families of physically abused children. *J. Clin. Child Psychol.* 19 313–328. 10.1207/s15374424jccp1904_3

[B66] Maureira Cid F. Flores Ferro E. (2017). Efectos del ejercicio físico sobre la atención: una revisión en los últimos años. *Rev. Activ. Física UCM* 18 73–83.

[B67] Merino-Navarro D. Díaz-Periánez C. (2020). Prevención y tratamiento del Covid-19 en la población pediátrica desde una perspectiva familiar y comunitaria. *Enfermería Clín.* [Epub ahead of print]. 10.1016/j.enfcli.2020.05.005 32425488 PMC7229975

[B68] Mitchell D. B. Hauser-Cram P. (2010). Early childhood predictors of mothers’ and fathers’ relationships with adolescents with developmental disabilities. *J. Intellect. Disabil. Res.* 54 487–500. 10.1111/j.1365-2788.2010.01268.x 20367745

[B69] Montero I. León O. G. (2007). A guide for naming research in psychology. *Int. J. Clin. Health Psychol.* 7 847–862.

[B70] Morrison J. Pikhart H. Ruiz M. Goldblatt P. (2014). Systematic review of parenting interventions in European countries aiming to reduce social inequalities in children’s health and development. *BMC Public Health* 14:1040. 10.1186/1471-2458-14-1040 25287010 PMC4203958

[B71] Mount N. Dillon G. (2014). Parents’ experiences of living with an adolescent diagnosed with an autism spectrum disorder. *Educ. Child Psychol.* 31 70–79.

[B72] Olhaberry M. Farkas C. H. (2012). Estrés materno y configuración familiar: estudio comparativo en familias chilenas monoparentales y nucleares de bajos ingresos. *Univ. Psychol.* 11 1317–1326.

[B73] Orben A. Tomova L. Blakemore S. J. (2020). The effects of social deprivation on adolescent development and mental health. *Lancet Child Adolesc. Health* 4 634–640. 10.1016/S2352-4642(20)30186-3 32540024 PMC7292584

[B74] Orgilés M. Fernández-Martínez I. Gonzálvez M. T. Espada J. P. (2016). Niños con síntomas de ansiedad por separación: un estudio de sus hábitos y problemas de sueño [Children with separation anxiety symptoms: a study of their habits and sleep problems]. *Ansiedad Estrés* 22 91–96. 10.1016/j.anyes.2016.10.004

[B75] Orgilés M. Morales A. Delvecchio E. Mazzeschi C. Espada J. P. (2020). Immediate psychological effects of the COVID-19 quarantine in youth from Italy and Spain. *Front. Psychol.* 11:579038. 10.3389/fpsyg.2020.579038 33240167 PMC7677301

[B76] Oronoz B. Alonso-Arbiol I. Balluerka N. (2007). A Spanish adaptation of the parental stress scale. *Psicothema* 19 687–692. 17959127

[B77] Ortega J. (2014). Estrés y evaluación Psicológica: una acercamiento teórico relacionado al concepto de resiliencia. *Anuario Investig.* 5 297–302.

[B78] Pérez Padilla J. Lorence Lara B. Menéndez Álvarez-Dardet S. M. (2010). Estrés y competencia parental: un estudio con madres y padres trabajadores. *Suma Psicol.* 17 47–57.

[B79] Pinto-Cortez C. (2014). Resiliencia Psicológica: una aproximación hacia su conceptualización, enfoques teóricos y relación con el abuso infantil. *Summa Psicol. UST* 11 19–33. 10.18774/448x.2014.11.129

[B80] Pisch M. Wiesemann F. Karmiloff-Smith A. (2019). Infant wake after sleep onset serves as marker for different trajectories in cognitive development. *J. Child Psychol. Psychiatry* 60 189–198. 10.1111/jcpp.12948 29989661

[B81] Prime H. Wade M. Browne D. T. (2020). Risk and Resilience in family well-being during the COVID-19 pandemic. *Am. Psychol.* 25 631–643. 10.1037/amp0000660 32437181

[B82] Putnick D. L. Bornstein M. H. Hendricks C. Painter K. M. Suwalsky J. Collins W. A. (2008). Parenting stress, perceived parenting behaviors, and adolescent self-concept in European American families. *J. Fam. Psychol.* 22 752–762. 10.1037/a0013177 18855511 PMC2575655

[B83] Quezada-Scholz V. E. (2020). Miedo y Psicopatología: la amenaza que oculta el COVID-19. *Cuadernos Neuropsicol.* 14 19–23. 10.2307/j.ctv1gd0vg5.5

[B84] Raikes H. A. Thompson R. A. (2005). Efficacy and social support as predictors of parenting stress among families in poverty. *Infant Ment. Health J.* 26 177–190. 10.1002/imhj.20044 28682501

[B85] Rajan A. M. John R. (2017). Resilience and impact of children’s intellectual disability on Indian parents. *J. Intellect. Disabil.* 21 315–324. 10.1177/1744629516654588 27329035

[B86] Ramón Fernández J. R. Llamas Salguero F. Gutiérrez Ortega M. (2019). Revisión bibliográfica y evolución del término resiliencia. *Rev. Educ. Hekademos* 26 40–47.

[B87] Respler-Herman M. Mowder B. A. Yasik A. E. Shamah R. (2011). Parenting beliefs, parental stres, and social support relationships. *J. Child Fam. Stud.* 21 190–198. 10.1007/s10826-011-9462-3

[B88] Romero-González M. Marín E. Guzmán-Parra J. Navas P. Aguilar J. M. Lara J. P. (2020). Relación entre el estrés y malestar psicológico de los padres y problemas emocionales y conductuales en niños preescolares con Trastorno del espectro autista. *Anales Pediatr.* 94 99–106. 10.1016/j.anpedi.2020.03.012 32402776

[B89] Rosenthal D. M. Ucci M. Heys M. Hayward M. Lakhanpaul M. (2020). Impacts of COVID-19 on vulnerable children in temporary accomodation in the UK. *Lancet Public Health* 5 241–242. 10.1016/S2468-2667(20)30080-3 PMC7270343 32243776

[B90] Saavedra Guajardo E. Villalta Paucar M. (2008). Medición de las características resilientes, un estudio comparativo en personas entre 15 y 65 años. *Liberabit* 14 31–40.

[B91] Sánchez-Teruel D. Robles-Bello M. A. (2014). Personalidad y resiliencia en un cuerpo especial de la Policía Nacional. *J. Work Organ. Psychol.* 30 75–81. 10.1016/j.rpto.2014.06.003

[B92] Sánchez-Teruel D. Robles-Bello M. A. (2015). Escala de Resiliencia 14 ítems (RS-14): propiedades Psicométricas de la Versión en Español. *Rev. Iberoamericana Diagn. Eval. Psicol.* 40 103–113.

[B93] Sandín B. Valiente R. M. García-Escalera J. Chorot P. (2020). Impacto psicológico de la pandemia de COVID-19: efectos negativos y positivos en población española asociados al periodo de confinamiento nacional. *Rev. Psicopatol. Psicol. Clín.* 25 1–22. 10.5944/rppc.27569

[B94] Serrano-Martínez C. (2020). Impacto emocional y crianza de menores de cuatro años durante el COVID-19. *Perifèria* 25 74–87. 10.5565/rev/periferia.735

[B95] Sun L. Sun Z. Wu L. Zhu Z. Zhang F. Shang Z. (2020). Prevalence and risk factors of accurate posttraumatic stress symptoms during the COVID-19 outbreak in Wuham, China. *medrxiv* [Preprint]. 10.1101/2020.03.06.20032425

[B96] Suriá Martínez R. (2013). Análisis comparativo de la fortaleza en padres de hijos con discapacidad en función de la tipología y la etapa en la que se adquiera la discapacidad. *Anuario Psicol. UB J. Psychol.* 43 23–37.

[B97] Suriá Martínez R. (2014). Discapacidad motora y Resiliencia: análisis en función de la edad, grado y etapa en la que se adquiere la discapacidad. *Rev. Siglo Cero* 250 6–18.

[B98] Suzuki K. Hiratini M. Mizukoshi N. Hayashi T. Inagaki M. (2018). Family resilience elements alleviate the relationship between maternal psychological distress and the severity of children’s developmental disorders. *Res. Dev. Disabil.* 83 91–98. 10.1016/j.ridd.2018.08.006 30145457

[B99] The Lancet Child Adolescent Health (2020). Prioritising children’s rights in the COVID-19 response. *Lancet Child Adolesc. Health* 4:279. 10.1016/S2352-4642(20)30172-3 32562626

[B100] Theule J. Wiener J. Tannock R. Jenkins J. M. (2013). Parenting stress in families of children with ADHD: a meta-analysis. *J. Emotion. Behav. Disord.* 21 3–17. 10.1177/1063426610387433

[B101] Tijeras Iborra A. (2017). *Estrés Parental e Impacto Familiar del Trastorno Del Espectro Autista: Factores Psicosociales Implicados.* Tesis Doctoral. Valencia: Repositorio Universidad de Valencia.

[B102] Tiwari G. K. Singh A. K. Parihar P. Pandey R. Sharma D. N. Rai P. K. (2020). Understanding the perceived health outcomes of children during COVID-19 pandemic. *medrxiv* [Preprint]. 10.22541/au.158958012.27449923

[B103] Trigueros R. Álvarez J. Aguilar-Parra J. M. Alcaraz M. Rosado A. (2017). Validación y adaptación española de la escala de resiliencia en el contexto deportivo (ERCD). *Psychol. Soc. Educ.* 9 311–324.

[B104] Tripp G. Schaughency E. A. Langlands R. Mouat K. (2007). Family interactions in children with and without ADHD. *J. Child Fam. Stud.* 16 385–400. 10.1007/s10826-006-9093-2

[B105] Vázquez C. Pérez-Sales P. Hervás G. (2008). “Positive effects of terrorism and posttraumatic growth: an individual and community perspective,” in *Trauma, Recovery and Growth: Positive Psychological Perspectives on Posttraumatic Stress*, eds Linley P. A. Joseph S. (Mahwah, NJ: Lawrence Erlbaum Associates), 63–91. 10.1002/9781118269718.ch4

[B106] Vela Llauradó E. Suárez Riveiro J. M. (2020). Resiliencia, satisfacción y situación de las familias con hijos/as con y sin discapacidad como predictores del estrés familiar. *Ansiedad Estrés* 26 59–62. 10.1016/j.anyes.2020.03.001

[B107] Villaceiros Durbán I. (2017). *Resiliencia Familiar: Un Acercamiento al Fenómeno de las Migraciones en la Triple Frontera Perú-Bolivia-Chile Desde la Perspectiva de Los Adolescentes.* Tesis Doctoral. Madrid: Universidad Pontificia Comillas.

[B108] Villalba Quesada C. (2003). El concepto de resiliencia individual y familiar. Aplicaciones en la intervención social. *Psychosoc. Intervent.* 12 283–299.

[B109] Viner R. M. Russell S. J. Croker H. Packer J. Ward J. Stansfield J. (2020). School closure and management practices during coronavirus outbreaks including COVID-19: a rapid systematic review. *Lancet Child Adolesc. Health* 4 397–404. 10.1016/S2352-4642(20)30095-X 32272089 PMC7270629

[B110] Wagnild G. (2009). A review of the resilience scale. *J. Nurs. Meas.* 17 105–113. 10.1891/1061-3749.17.2.105 19711709

[B111] Wagnild G. Young H. M. (1993). Development and psychometric evaluation of the resilience scale. *J. Nurs. Meas.* 1 165–178. 7850498

[B112] Wang C. Pan R. Wan X. Tan Y. Xu L. Ho C. S. (2020). Immediate psychological responses and associated factors during the initial stage of the 2019 coronavirus disease (COVID-19) epidemic among general population in China. *Int. J. Environ. Res. Public Health* 17:1729. 10.3390/ijerph17051729 32155789 PMC7084952

[B113] Wang P. Michaels C. A. Day M. S. (2011). Stresses and coping strategies of chinese families with children with autism and other developmental disabilities. *J. Autism. Dev. Disord.* 41 783–795. 10.1007/s10803-010-1099-3 20859670

[B114] Windle G. (2011). What is resilience? A review and concept analysis. *Rev. Clin. Gerontol.* 21 152–169. 10.1017/S0959259810000420

[B115] World Health Organization [WHO] (2020). *Spotlight on Adolescent Health and Well-Being. Findings from the 2017/2018 Health Beaviour in School-Aged Children (HBSC) Survey in Europe and Canada.* Geneva: World Health Organization.

[B116] Zarzaur B. L. Bell T. M. Zanskas S. A. (2017). Resiliency and quality of life trajectories after injury. *J. Trauma Acute Care Surg.* 82 939–945. 28230626 10.1097/TA.0000000000001415 PMC5753399

